# Novel bidirectional traction technique for tension-regulated large defect closure: a case report

**DOI:** 10.1055/a-2793-3571

**Published:** 2026-03-02

**Authors:** Weixing Yang, Tao Hao, Xiaowei Tang, Xiaolin Zhong, Hao Li, Muhan Lü

**Affiliations:** 1 556508 Department of Gastroenterology, The Affiliated Hospital, Southwest Medical University, Luzhou, China; 2 Department of Gastroenterology, Tianfu Affiliated Hospital, Southwest Medical University, Meishan, China

Endoscopic submucosal dissection (ESD) for large mucosal defects presents a critical challenge in achieving secure closure, a factor that significantly influences postoperative recovery and clinical outcomes 1 . Although several techniques have been developed to facilitate defect closure, such as the clip-with-line method and the overstitch endoscopic suturing system, the management of extensive defects remains hampered by technical complexity, substantial financial cost, and notable postoperative risks 2 3 . Herein, a novel traction-assisted technique was designed to achieve reliable mucosal apposition under technically demanding conditions ( Video 1 ).

The steps for defect closure using the novel strategy.Video 1

**Fig. 1:**
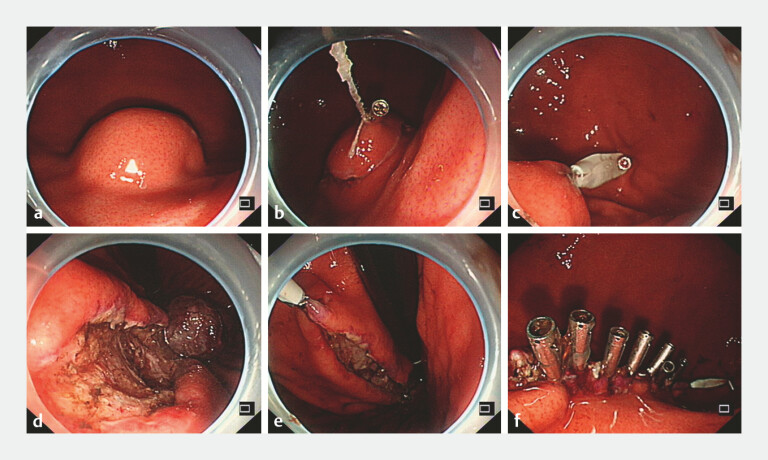
Endoscopic views demonstrating the novel bidirectional traction technique for the closure of a large post-ESD defect. **a** A 2.7 × 2.1 cm gastrointestinal stromal tumor was located on the posterior gastric wall. **b** The oral-traction point was established by grasping and anchoring a preserved tumor fragment to the proximal mucosa with a clip. **c** Anal counter-traction was applied to minimize tissue inversion. **d, e** The defect was sequentially reconfigured into a spindle shape and closed along the long axis with metallic clips. **f** The complete linear apposition of the mucosal edges. ESD, endoscopic submucosal dissection.

A 62-year-old woman with a 2.7 × 2.1 cm gastrointestinal stromal tumor located on the posterior gastric wall underwent en bloc ESD. The resulting defect measured approximately 5 × 4 cm with a near-circular configuration ( Fig. 1 **a** ). Given the extensive size and suspected full-thickness involvement, direct closure using conventional metallic clips was deemed unfeasible. An innovative stepwise closure strategy was therefore employed.

Step 1: Establishment of the oral traction point: Rather than completing full resection, a portion of the tumor at the oral side was deliberately preserved to serve as a native traction anchor. This was grasped and fixed to normal mucosa oral to the defect using a clip, generating sustained oral-directed vector forces to tension the edge ( Fig. 1 **b, c** ).

Step 2: Establishment of the anal-traction point: On the anal side of the defect, internal traction was applied by grasping the mucosal edge and exerting a force in the anal and luminal directions. This maneuver generated effective counter-traction, thereby maintaining adequate tissue tension and minimizing undesirable outward inversion (everting towards the serosal side) of the mucosal margin ( Fig. 1 **d, e** ).

Step 3: Sequential linear closure: Through these bidirectional forces, the originally circular defect was geometrically reconfigured into a spindle-shaped defect with reduced circumference and well-opposed edges, preventing tissue inversion ( Fig. 1 **f** ). Using metallic clips, sequential closure was performed from one apex along the long axis of the reconfigured defect, resulting in linear apposition without the suture material.

**Fig. 2:**
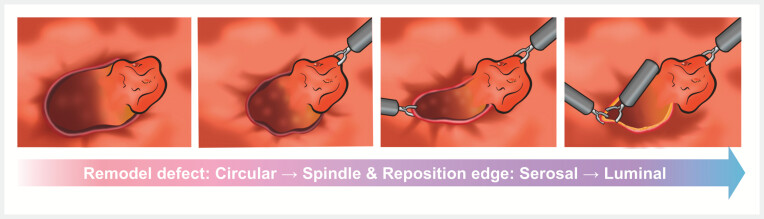
A schematic diagram of the bidirectional traction technique.

As illustrated in the schematic ( Fig. 2 ), this technique utilizes purposeful traction anchors and controlled bidirectional forces to actively remodel the defect into a geometry amenable to efficient closure. This approach addresses two fundamental issues: (1) linearization reduces the defect circumference and tension, facilitating clips placement; (2) traction-assisted edge alignment minimizes tissue inversion, thereby reducing the risk of improper healing and delayed perforation. Relying solely on conventional metallic clips, this method is both technically accessible and cost-effective. It offers a promising new strategy for managing challenging post-ESD defects.

Endoscopy_UCTN_Code_TTT_1AO_2AO
